# Low penetrance in facioscapulohumeral muscular dystrophy type 1 with large pathological D4Z4 alleles: a cross-sectional multicenter study

**DOI:** 10.1186/s13023-014-0218-1

**Published:** 2015-01-21

**Authors:** Emmanuelle Salort-Campana, Karine Nguyen, Rafaelle Bernard, Elisabeth Jouve, Guilhem Solé, Aleksandra Nadaj-Pakleza, Julien Niederhauser, Estelle Charles, Elisabeth Ollagnon, Françoise Bouhour, Sabrina Sacconi, Andoni Echaniz-Laguna, Claude Desnuelle, Christine Tranchant, Christophe Vial, Frederique Magdinier, Marc Bartoli, Marie-Christine Arne-Bes, Xavier Ferrer, Thierry Kuntzer, Nicolas Levy, Jean Pouget, Shahram Attarian

**Affiliations:** AP-HM, Reference Center of Neuromuscular Disorders and ALS, Timone University Hospital, Aix-Marseille University, 264 rue Saint-Pierre, Marseille, Cedex 05 13385 France; Aix Marseille Université - Inserm UMR_S 910 Medical Genetics and Functional Genomics, Marseille, France; AP-HM, Department of Medical Genetics, Timone University Hospital, Marseille, France; CIC-UPCET, Timone University Hospital, AP-HM, UMR CNRS Aix-Marseille University 6193, Marseille, France; Reference Center of Neuromuscular Disorders, CHU of Bordeaux, Pessac, France; Centre de Référence des Maladies Neuromusculaires Nantes-Angers, Service de Neurologie, CHU d’Angers, Angers, France; Nerve-Muscle Unit, Department of Clinical Neurosciences, Lausanne University Hospital (CHUV), Lausanne, Switzerland; Croix-Rousse Hospital, Lyon, France; Electroneuromyography and Neuromuscular Department, GHE Neurologic hospital, Lyon, Bron Cedex 69677 France; Neuromuscular Disease Specialized Center, Nice University Hospital, Nice, France; Reference Center of Neuromuscular Disorders, Neurology Department, Hautepierre Hospital, Strasbourg, France; Reference Center of Neuromuscular Disorders, CHU of Toulouse, Toulouse, France

**Keywords:** Facioscapulohumeral muscular dystrophy, Penetrance, FSHD1, D4Z4

## Abstract

**Background:**

Facioscapulohumeral muscular dystrophy type 1(FSHD1) is an autosomal dominant disorder associated with the contraction of D4Z4 less than 11 repeat units (RUs) on chromosome 4q35. Penetrance in the range of the largest alleles is poorly known. Our objective was to study the penetrance of FSHD1 in patients carrying alleles ranging between 6 to10 RUs and to evaluate the influence of sex, age, and several environmental factors on clinical expression of the disease.

**Methods:**

A cross-sectional multicenter study was conducted in six French and one Swiss neuromuscular centers. 65 FSHD1 affected patients carrying a 4qA allele of 6–10 RUs were identified as index cases (IC) and their 119 at-risk relatives were included. The age of onset was recorded for IC only. Medical history, neurological examination and manual muscle testing were performed for each subject. Genetic testing determined the allele size (number of RUs) and the 4qA/4qB allelic variant. The clinical status of relatives was established blindly to their genetic testing results. The main outcome was the penetrance defined as the ratio between the number of clinically affected carriers and the total number of carriers.

**Results:**

Among the relatives, 59 carried the D4Z4 contraction. At the clinical level, 34 relatives carriers were clinically affected and 25 unaffected. Therefore, the calculated penetrance was 57% in the range of 6–10 RUs. Penetrance was estimated at 62% in the range of 6–8 RUs, and at 47% in the range of 9–10 RUs. Moreover, penetrance was lower in women than men. There was no effect of drugs, anesthesia, surgery or traumatisms on the penetrance.

**Conclusions:**

Penetrance of FSHD1 is low for largest alleles in the range of 9–10 RUs, and lower in women than men. This is of crucial importance for genetic counseling and clinical management of patients and families.

## Background

Facioscapulohumeral muscular dystrophy (FSHD) is an autosomal dominant disorder with variable severity and inter- and intra-familial heterogeneity [1,2]. FSHD is characterized by a highly selective and asymmetrical pattern of muscle involvement. For two decades, the molecular diagnosis of FSHD type 1 (FSHD1) has been supported by the evidence of a heterozygous contraction of the D4Z4 repeat array from 1 to 10 repeat units (RUs) on 4q35 [3].

D4Z4 contraction is considered to be pathogenic if it occurs on a specific chromosomal background, i.e., (i) the presence of the 4A (159/161/168) haplotype and (ii) a single nucleotide polymorphism that creates a polyadenylation site (PAS) for the distal DUX4 transcript [4-6]. The unique association of FSHD1 with a specific haplotype remains controversial as some FSHD1 patients carry a D4Z4 contraction without the common 4A161PAS haplotype [7]. The pathological cut-off is conventionally determined at 10 RUs. Most FSHD1 patients carry 1 to 8 RUs on one allele. Large studies provided data on expressivity and penetrance in this range [8,9]. However, few studies reported the phenotypic spectrum associated with the upper pathological alleles (9–10 RUs). It was suggested that these patients present with a later and milder phenotype and a lower penetrance [10-14]. A better knowledge of penetrance in this range is necessary for improving genetic counseling, particularly in the context of predictive testing and prenatal diagnosis in FSHD families.

In our study, we aimed to estimate the penetrance in a large group of FSHD1 patients with alleles of 6–10 RUs. In addition, we evaluated the influence of sex, age, and several environmental factors on clinical expression of the disease.

## Methods

A cross-sectional multicenter study was conducted in six French and one Swiss neuromuscular centers. Among the FSHD cases followed in the reference centers from 2007 to 2009, patients carrying a contracted D4Z4 array with an estimated size of 6 RUs (27 kb) to 10 RUs (40 kb) and fulfilling the diagnostic criteria defined by the European Expert Group on FSHD [15] were selected to constitute the index case (IC) group. The second group included volunteer at-risk relatives with various degrees of kinship. For ethical considerations, we did not include patients less than 18 years of age or pregnant women.

At each center, patients and relatives were examined by a pluridisciplinary team that included a neurologist with neuromuscular disorder expertise, a geneticist and a physiotherapist. All clinical data were collected and blood was sampled for genetic analysis during a single visit.

All subjects enrolled in the study gave informed consent to participate. This study received the approval of the local ethic committee (Comité de Protection des Personnes Sud Mediterranée I).

### Clinical and functional evaluation

For each IC or relative, we recorded the following: medical history, self-evaluation of physical activity, pedigree, clinical examination, functional assessment, and manual muscular test results. ICs and relatives were asked for information pertaining to physical activity (self evaluation of physical activity), number of trauma requiring an immobilization of one week or more,, number of exposures to and types of anesthesia received, exposure to tobacco (number of pack-years of cigarettes), alcohol intake (average number of alcohol units per day and per week), and the use of any drugs. The first symptom experienced and the age of onset were recorded for ICs only. We did not record the age of onset in relatives because some of them had never been examined before and did not want their clinical status to be revealed. Therefore, we established the age at examination as the age of onset for clinically affected relatives. Neurologists examined the presence or absence of scapular winging, facial, limbs and thoraco-abdominal muscle weakness, selective involvement and asymmetry of muscle weakness. At the end of clinical examination, the neurologist blinded to the genetic results determined if the relative was clinically affected or not. The guidelines were to consider that a relative was clinically affected if there was facial and/or scapular fixator weakness; and there was absence of atypical signs suggesting an alternative diagnosis (including extraocular, masticatory, pharyngeal, or lingual muscle weakness and cardiomyopathy) [10,15].

### Genetic testing

Informed consent was obtained for every enrolled subject. In the relatives group, each participant was asked if he wanted to be informed about his own genetic results. Subjects who wished to be informed were given their genetic result by the geneticist during a genetic counseling session at the end of the study. The genetic result was validated by a second analysis on a new DNA sample. Each patient’s blood sample was coded and anonymized.

All genetic analyses were performed at the Laboratory of Molecular Genetics in the Department of Medical Genetics in Marseille. DNA was prepared from isolated lymphocytes according to standard procedures. Restriction endonuclease digestion was performed and digested DNA was separated by pulsed-field gel electrophoresis (PFGE) in a 1% agarose gel. Allele sizes were estimated by Southern blot hybridization with a radiolabeled p13E-11 probe using 7 μg of *Eco*RI and *Eco*RI/*Bln*I digested genomic DNA. In our laboratory practice and according to interlaboratory quality assessment, we consider that the pathological cut-off at 10 RUs corresponds to an *Eco*RI fragment of 40 kb. Thus, the range of 6–10 RUs corresponds to fragments from 27-40 kb. The molecular results in our diagnostic laboratory are provided in RUs. The 4qA/4qB allelic variants were identified using 7 μg of *Hind*III digested DNA, PFGE electrophoresis, and Southern blot hybridization with radiolabeled probes for qA/qB according to standard procedures. The IC group was analyzed with *Eco*RI/p13E-11, *Eco*RI*/Bln*I*/*p13E-11, and *Hind*III/qAqB while the relatives group was analyzed by comparing their *Eco*RI*/Bln*I*/*p13E-11 pattern with that of the IC in each family. We considered as “carriers” the relatives harboring the contracted allele previously identified in the IC. We did not perform the analysis of the SSLP-PAS sequence, as the recent literature indicates that only 50% of FSHD1 patients carry the 4A161PAS permissive haplotype associated with the D4Z4 contraction. Thus, we did not consider this parameter as mandatory for molecular diagnosis [16].

### Statistical analyses

To examine group comparability, we compared the age and sex of ICs, clinically affected carriers, clinically unaffected carriers, and non carriers. To study the influence of age, sex, and environmental factors on the risk of being clinically affected, we compared clinically affected versus clinically unaffected carriers.

For all mean comparisons, the Student’s t-test or analysis of variance were used. Frequencies were compared using a chi-square test. When necessary, nonparametric tests (Mann–Whitney or Kruskal–Wallis tests) or Fisher exact tests were used. Differences were considered significant for all analyses when the P values were lower than 0.05.

The risk of developing symptoms of the disease considering age, sex and number of RUs was estimated for clinically affected patients (IC and clinically affected carriers) versus clinically unaffected carriers using the multivariate logistic regression model. Odds ratios (ORs) and 95% confidence intervals (CIs) were calculated for age (continuous variable), sex and number of RUs classified into two categories (6–8 RUs, 9–10 RUs).

To estimate the penetrance, the primary criterion was the clinical status established by neurologists blinded with regard to genetic status. Penetrance was defined as the ratio between the number of carriers considered as clinically affected and the total number of carriers. In order not to underestimate the penetrance by taking in account the at-risk relatives only, we estimated the penetrance as a range between two figures including or excluding the ICs.

The Kaplan–Meier survival analysis was used for ICs and clinically unaffected carriers to estimate the age-specific cumulative first symptom incidence with the corresponding 95% CIs. For each IC, the time from birth to the earliest declarative age at onset of symptoms was collected. For clinically unaffected carriers, we considered the age at the time of the clinical evaluation.

## Results

**Figure 1:**
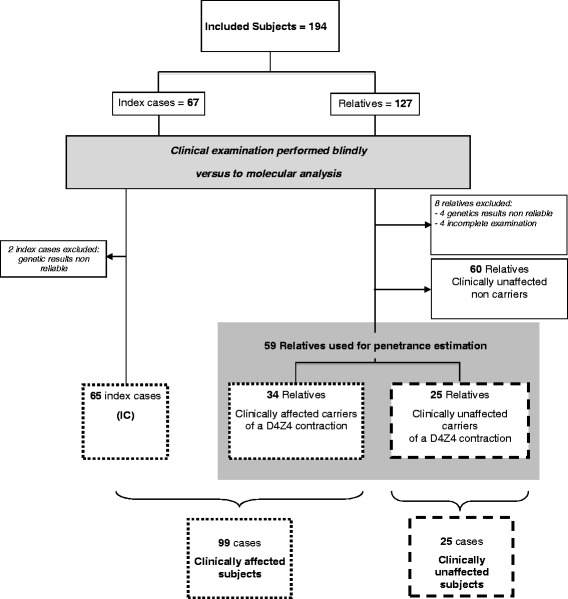
**Repartition of subjects included in the study.**

184 subjects were included in the study, 65 ICs and 119 relatives (Figure 1). Forty-four families encompassed less than 4 individuals and 21 families encompassed between 4 and 9 individuals. All ICs harbored a 4qA contracted allele. Among the 119 relatives, 59 were carriers of the D4Z4 contraction identified in the IC, and 60 were non carriers. Among the 59 relatives, 34 were clinically affected carriers and 25 were unaffected carriers.

Overall, 124 were carriers of the contraction, of which 99 individuals were clinically affected (65 ICs and 34 affected relatives).

### Demographic characteristics of the subjects

Descriptive statistics of age and sex are described in Table 1. Of the 99 clinically affected individuals (ICs and affected carriers), there were 62 men and 37 women. In the remaining unaffected carrier patients group, there were significantly more women (18 of 25, p = 0.002). The age of the patients at the time of clinical evaluation did not significantly differ between ICs, affected carriers, unaffected carriers, and non carriers. Table 2 summarizes the phenotype observed in the index cases and the affected carriers.

**Table 1:** **Descriptive statistics of age and sex**

	**ICs n (%)**	**Affected carriers n (%)**	**Unaffected carriers n (%)**	**Non carriers n (%)**	**P value**
	65	34	25	60	
Gender					0.002
Male	44 (68%)^a,b^	18 (53%)	7 (28%)^a^	24 (40,%)^b^	
Female	21 (32%)	16 (47%)	18 (72%)	36 (60%)	
Age, years (mean ± standard deviation)	51.2 ± 14.9	50.5 ± 17.4	47.6 ± 19.5	46.9 ± 17.4	0.482

Overall age comparison: Kruskall–Wallis test p = 0.482. Overall gender comparison: chi square test p = 0.002. Significant pairwise comparisons: ^a^ICs versus unaffected carriers p = 0.0007 and ^b^ICs versus non carriers p = 0.001.

**Table 2:** **Phenotype in index cases and affected carriers at the time of examination**

	**ICs**	**Affected carriers**
	**n (%)**	**n (%)**
Facial weakness	49 (75%)	20 (59%)
Scapular weakness	65 (100%)	32 (94%)
Pectoral weakness	55 (85%)	20 (59%)
Abdominal weakness	51 (78.5%)	20 (59%)
Asymmetry	49 (75%)	23 (68%)
Steppage	31 (48%)	2 (6%)

In the IC group (n = 65), the mean age at onset was 30.0 years (±16.5). Thirty-four patients (52.3%) had developed symptoms before 30 years of age. The mean age at diagnosis was 44.2 years (±15.9). The mean disease duration at inclusion was 7.2 years (±6.9). The mean diagnosis delay was 14.2 years (ranging 1–49 years). The type of first symptoms experienced by patients was scapulo-humeral weakness in 40 patients (61.5%), facial weakness in 3 patients (4.6%), lower limb weakness in 18 patients (27.7%), muscle pain in 2 patients (3.0%), clumsiness in one patient (1.5%) and not informed in one patient. When comparing the, ICs carrying 6–8 RUs (n = 49) and those carrying 9–10 RUs (n = 16), we found a higher proportion of patients developing symptoms before 30 years in the 6–8 RUs subgroup (59.2%) than in the 9–10 RUs subgroup (31.3%), although it did not reach statistical significance (p = 0.052). Of 7 patients in the IC group (2 men and 5 women) who were wheelchair-bound (mean age 60.9 years), six of them belonged to the 6–8 RUs subgroup.

The relatives were divided into the two subgroups by number of RUs: 6–8 RUs (n = 40) and 9–10 RUs (n = 19).

### Study of penetrance

Overall, among the 124 carriers, 99 subjects were clinically affected (65 ICs and 34 relatives), which leads to estimate the penetrance of the disease in the whole group of 6–10 RUs at 57% (if ICs are excluded) or 79% (if ICs are included). In the 6–8 RUs subgroup, penetrance varied from 62 to 83% whereas in the 9–10 RUs subgroup, it ranged from 47% to 71%. We found a higher proportion of clinically unaffected versus clinically affected carriers in the 9–10 RUs group than in the 6–8 RUs group, although it did not reach the statistical significance.

**Figure 2:**
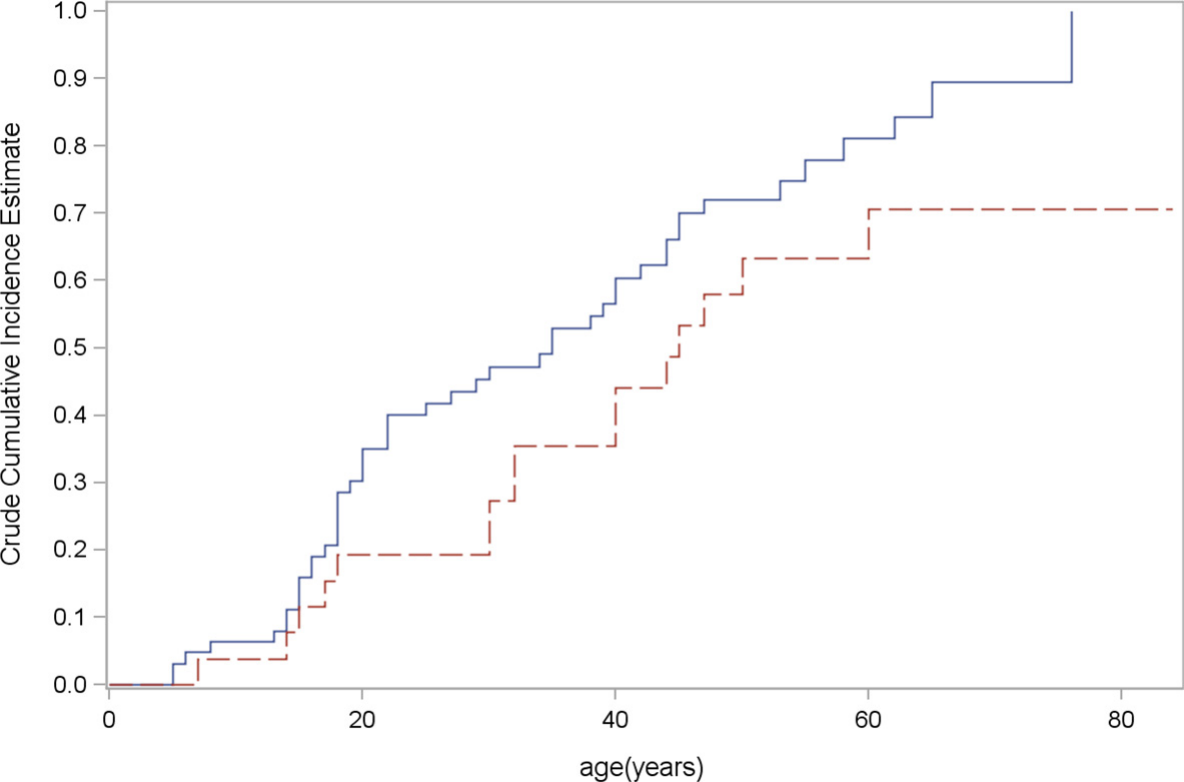
**Age-related cumulative risk of being clinically affected according to the number of RUs.** Estimates obtained on 65 IC and 25 unaffected carriers using the Kaplan-Meier analysis. Blue line refers to 6–8 RUs; Red line refers to 9–10 RUs. Carriers 6–8 RUs versus 9–10 RUs, Log-Rank test P = 0.0584.

An age-related cumulative frequency of penetrance was estimated using the Kaplan–Meier method on the data obtained from 65 ICs and 25 unaffected carriers (Figure 2 and Table 3). In the 6–8 RUs subgroup, the risk of developing motor impairment was 35% by age 20, 60% by age 40 and 81% by age 60, whereas in the 9–10 RUs subgroup, the risk was 19% by age 20, 44% by age 40, and 71% by age 60.

**Table 3:** **Estimates of the age-specific cumulative risk using Kaplan–Meier analysis**

**Age**	**6–8 RUs**		**9–10 RUs**	
**(years)**	**Risk**	**95% CI**	**Risk**	**95% CI**
20	35.1	(25.0; 49.1)	19.2	(8.7; 42.3)
30	47.2	(36.1; 61.6)	27.3	(14.5; 51.4)
40	60.4	(48.9; 74.5)	44.0	(28.2; 68.7)
50	72.0	(61.1; 85.0)	63.3	(46.1; 86.9)
60	81.1	(70.2; 93.7)	70.6	(52.7; 94.5)
70	89.5	(78.9; 100)		

### Influence of age, sex, environmental factors and degree of kinship on penetrance

In order to evaluate the influence of age, sex, and environmental factors on penetrance, we compared the affected and the unaffected carriers. We found no difference with the age criterion. Seventy two percent of the unaffected carriers (18/25) were women, although it did not reach statistical significance (p = 0.055). There was no difference of smoking habits or intake of alcohol between the two groups. Regarding medical history (high blood pressure, cardiovascular diseases, diabetes mellitus, cancer, thyroid disorders, trauma, and anesthesia), level of physical activity, and exposure to medication (analgesics, anti-inflammatory, cholesterol-lowering drugs, or anti-depressants), there was no difference either. We did not find any effect of the degree of kinship.

The risk of developing FSHD between clinically affected patients (ICs and affected carriers, n = 99) and clinically unaffected carriers (n = 25) was evaluated by logistic regression analysis. There was no significant effect of age on the risk of developing motor impairment (OR = 1.02, CI = 0.99–1.05). The risk of developing symptoms of FSHD was 4.6-fold higher (p = 0.0041; OR = 4.6, CI = 1.6–12.8) for men than for women, suggesting a significant effect of sex in the disease. Regarding the influence of the number of RUs on penetrance, the risk of developing symptoms of FSHD was not significantly increased in patients with 6–8 RUs versus in patients with 9–10 RUs (p = 0.72; OR = 2.01, CI = 0.74–5.50).

## Discussion

We have conducted a cross-sectional multicenter study of the penetrance in FSHD1 patients carrying contracted alleles of 6–10 RUs. In this range, the penetrance was estimated to be 57% (if ICs are excluded) or 79% (if ICs are included). The penetrance was lower in patients with 9–10 RUs (47% if ICs are excluded or 71% if ICs are included) than in those with 6–8 RUs (62% if ICs are excluded or 83% if ICs are included).

In the premolecular era, the penetrance of FSHD1 was considered to be age-related and almost complete at age 20 [17]. When including D4Z4 analyses, the penetrance was shown to be incomplete but higher than 80% [13,14,18,19]. More recently, the penetrance was shown to be lower than expected. In a retrospective study of 85 Japanese families, including sporadic and familial cases, Goto et al. [20] observed a penetrance of 59%. Of late, a Greek survey of 133 patients estimated that the penetrance ranged from 50 (ICs excluded) to 77% (ICs included) in FSHD1 patients carrying a contracted allele ≤38 kb [9]. In addition, in a large Italian cohort, Ricci et al. estimated the penetrance in patients with alleles of 1–8 RUs [8]. The estimated risk of developing motor impairment by age 60 for relatives carrying 1–3 RUs and 4–8 RUs was 96.2% and 71.5%, respectively. These results suggest an inverse relationship between penetrance and size of the contracted allele.

In the range of the largest pathological alleles, penetrance data are scarce to date. Clinical reports have suggested that the penetrance in this range is low, but it has never been calculated through large family studies [10,11,21,22]. Our cohort is the largest one including FSHD1 families with contracted alleles in the upper range of pathological alleles (9–10 RUs). We showed that the age-related cumulative risk of being clinically affected was lower in patients with 9–10 RUs than in patients with 6–8 RUs (70% versus 81% at age 60), although the difference was not statistically significant. To compare, in the Italian survey, the risk of being clinically affected in patients with 7–8 RUs was lower than in patients with 4–6 RUs (63% versus 71% at age 60) [8]. Our study suggests the same inverse relationship between penetrance and size of the contracted allele in the range 9–10 RUs, as observed for alleles smaller than 8 RUs.

In our study, the mean age of onset (30 yo) was higher than reported in previous studies, ie 16 years [23] and 23 years [24]. In our cohort, only 52% of ICs had developed symptoms before 30, contrasting with the 70% reported elsewhere [25]. Several studies in FSHD1 have shown a significant correlation between age at onset and EcoRI fragment size [26]. All the previous studies included patients with alleles <6 RUs while we excluded them from our cohort. Accordingly, our data suggest that the disease onset in patients with the largest alleles is later than in those with the shortest alleles. In agreement with previous studies, we found a significant effect of sex on FSHD1 penetrance, with women being less clinically affected than men [2,8,14,18,19]. Our data confirm the influence of sex, even in alleles in the upper range.

The concordance between the clinical status determined by the neurological examination and the results of the genetic analysis was complete in this study, emphasizing the high predictive value of clinical expertise in accurate FSHD diagnosis. Regarding the mode of onset of the disease, the first symptom was scapulohumeral weakness in 61% of patients, as in previous studies [8,9]. The onset in the lower limbs was higher in our series (28%) than previously reported (11.7 to 16.7%) [8,9]. Furthermore, only 10.7% of our ICs were wheelchair-bound (mean age of 50 years; mean disease duration of 7 years) while previous studies reported that approximately 20% of FSHD1 patients become wheelchair-bound by the age of 50 [9,27,28]. We hypothesize that in the range of the upper alleles, the clinical course of the disease is less severe in line with the fact that the most severe phenotypes usually result from the shortest alleles (1–3 RUs) [29,30].

In addition, we searched for an environmental effect that could influence the FSHD1 penetrance. We did not find any effects of drugs, anesthesia, surgery or traumatisms on the penetrance. Nevertheless, the number of patients included in this study was too small for epidemiological studies. Further investigations in large cohorts are needed to exclude the effect of environmental factors on FSHD. Besides, previous studies suggested that epigenetic mechanisms or modifier genes influence the clinical expression of FSHD [31].

Recently, a novel gene called SMCHD1 has been identified as causative in FSHD without D4Z4 contraction (FSHD2), and has also been proposed as a modifier in FSHD1 [32,33]. In our study, penetrance could have been influenced by epigenetic factors, which should be further explored.

## Conclusion

In conclusion, penetrance is low in patients with alleles in the upper range and lower in women than men. In this range, the age of onset is later and the course of the disease is less severe. Knowledge of penetrance and expressivity of FSHD1 is of crucial importance for genetic counseling and clinical management of patients and families. Our findings indicate that great caution must be taken concerning predictive and prognostic information provided to FSHD1 carriers of the largest alleles. Deeper investigations of genotype-phenotype correlations are ongoing in our cohort in order to provide a precise characterization of this patient subgroup.

## Abbreviations

FSHD: Facioscapulohumeral muscular dystrophy
FSHD1: Facioscapulohumeral muscular dystrophy type 1
IC: Index case
RUs: Repeat units
